# No association between the *PREP *gene and lithium responsive bipolar disorder

**DOI:** 10.1186/1471-244X-7-9

**Published:** 2007-02-26

**Authors:** Firoza Mamdani, Adolfo Sequeira, Martin Alda, Paul Grof, Guy Rouleau, Gustavo Turecki

**Affiliations:** 1 Douglas Hospital Research Centre, McGill University, Montreal, Quebec, Canada; 2 Department of Psychiatry, McGill University, Montreal, Quebec, Canada; 3 Mood Disorders Clinic of Ottawa, Ottawa, Ontario, Canada; 4 Université de Montréal, Montréal, Quebec, Canada

## Abstract

**Background:**

Bipolar disorder (BD) is a major psychiatric condition that commonly requires prophylactic and episodic treatment. Lithium (Li) has been used for over 40 years now as an effective prophylactic agent. Response to Li treatment seems to be, at least in part, genetically determined. Although we ignore how Li specifically prevents mood episodes, it has previously been suggested that Li exerts an effect on the phosphoinositide pathway, and more recently, it has been proposed that Li may modulate prolyl endopeptidase (*PREP*).

**Methods:**

In this study we carried out an association study looking at the *PREP *gene, located on ch 6q22. Five intronic single nucleotide polymorphisms (SNP), three coding SNPs and one SNP in the 5' UTR were investigated for their frequency in a BD sample of 180 excellent Li responders, 69 Li nonresponders and 126 controls. Genotyping was carried out using the SNaPshot reaction from Applied Biosystems, which is a modified fluorescent single base pair extension procedure.

**Results:**

Following correction for multiple testing, no significant genotypic, allelic or estimated haplotypic differences were found between responders and nonresponders or between BD patients and controls.

**Conclusion:**

*PREP *is an interesting candidate gene to investigate in genetic studies of BD, but our findings do not support the hypothesis that genetic variation in this gene plays a major role in the etiology of BD or Li response.

## Background

Lithium has been one of the most widely used and best characterized prophylactic treatments for bipolar disorder (BD). Even though the therapeutic effects of lithium have been well documented for many years, its mechanism of action remains unknown. Several plausible pathways have been hypothesized to be influenced by lithium; the phosphoinositide pathway being one [1]. It has been demonstrated that lithium inhibits two key enzymes involved in the regeneration of free inositol. One of these is inositol monophosphatase (IMPase), which is the enzyme responsible for the conversion of inositol monophosphate (IMP) to free inositol. The other one is inositol polyphosphatase (IPP), which is the enzyme catalyzing the transformation of inositol 1,4-bisphosphate to IMP [2,3]. The effects of the lithium induced inhibition results in the decrease of phosphatidyl inositol-4,5-bisphosphate (PIP2). This subsequently causes a decrease in the downstream signaling molecules, inositol-1,4,5-trisphosphate (IP3) and diacylglycerol (DAG) which are produced through the phospholipase C catalyzed breakdown of PIP2. This process initiates a dampening of cellular response to second messengers [4]. It is for this reason that genes involved in this pathway are good candidates for investigation

One such gene is prolyl endopeptidase or prolyl oligopeptidase (*PREP*). Prolyl endopeptidase has been shown to be highly active in the brain, especially in the frontal cortex and in the limbic system [5,6]. *PREP *is a cytosolic serine protease that has been shown to cleave neuropeptides made up of 30 residues or less, for instance thyrotropin-releasing hormone, arginine vasopressin, and others [7]. Investigating possible mechanisms of lithium's action in Dictyostelium, an amoebae used as a model system for molecular genetic studies, Williams and colleagues (1999) reported that lithium resistant mutants lacked the *PREP *gene and showed high levels of IP3. This increase is likely the result of the inability of Dictyostelium to dephosphorylate IP3 into IP2. Thus, these results provided experimental evidence for a link between the lack of *PREP *expression and increased IP3 levels. In addition, this data adds evidence to the implication of the inositol second-messenger system in BD and lithium's mode of action. These findings are in accordance with a previously reported association between abnormal *PREP *activity and bipolar disorder/major depression [8]. Recently, Williams et al. [9] added new results suggesting a common, underlying mechanism via inositol depletion for lithium, valproic acid and carbamazepine, all commonly used mood stabilizers for bipolar disorder. The authors showed that Dictyostelium resistance in mutants to these drugs was conferred by *PREP*, further suggesting a possible role of prolyl endopeptidase as a mediator of these drugs' mood stabilizing action through the inositol system.

Interestingly, the *PREP *gene is located on chromosome 6q22, a region that has been linked to BD in several studies [10-12] and most recently in Portuguese families [13-15], thus providing added incentive to investigate genes in this chromosomal region.

The purpose of this paper was to investigate variation at the *PREP *gene in a sample of BD patients with either excellent lithium response or nonresponse.

## Methods

### Subjects

The sample used for this study was composed of 251 patients with only a diagnosis of BD. Of these patients, a hundred forty-five patients were females and 104 were male, with a mean age of 47.91 ± 14.09 years and a mean age of onset of 26.15 ± 9.71. Of the cases 180 were responders (LiR) and 69 were nonresponders (LiNR) to long term lithium treatment. All psychiatric assessments were carried out by experienced clinicians using the SADS-L structured interviews, and the RDC and DSM-IV criteria. Excellent lithium response was established as described previously [16] and summarized in Table 1, with the number of pretreatment episodes (± standard deviation) being 8.04 ± 5.75 and treatment length being 13.25 ± 7.26 years. Controls included in this study were 126 healthy, psychiatrically normal individuals (mean age 48.17 ± 11.84) that were assessed using the same criteria as for patients. Prior to inclusion in the study, all individuals provided written informed consent. This study was approved by the local review boards of each institution. All subjects included in this study were Caucasian of European origin that were recruited in centers that are part of the International Group for the Study of Lithium (IGSLi), a collaborative group of specialized clinics in Canada, Austria, Czech Republic, Denmark, Germany, and Sweden. To ensure diagnostic uniformity across the clinics all patients were subsequently evaluated by the same senior clinician (PG).

**Table 1:** Criteria used to diagnose bipolar patients as excellent lithium responders

Each patient must meet criteria **A**, **B **and **C**	
**A.**	**Diagnosis of primary episodic bipolar disorder based on the SADS-L (lifetime version) interview and Research Diagnostic Criteria (RDC).**
**B.**	**High Recurrence Risk**
*either*	**B1**: five or more episodes prior to lithium treatment
*or*	**B2**: four episodes prior to lithium; of these two or more during the 2 years preceding lithium treatment
*or*	**B3**: three episodes prior to lithium, plus one more within 12 months after lithium discontinuation
**C.**	**Unequivocal Lithium Response**
	**C1**: No recurrence requiring additional biological intervention (ECT, antidepressants, neuroleptics) during the entire observation time on lithium monotherapy
*and*	**C2**: minimum period of observation of 3 years
*and*	**C3**: average plasma lithium concentration over 0.6 mEq L^-1^

### DNA analysis

**Figure 1:**
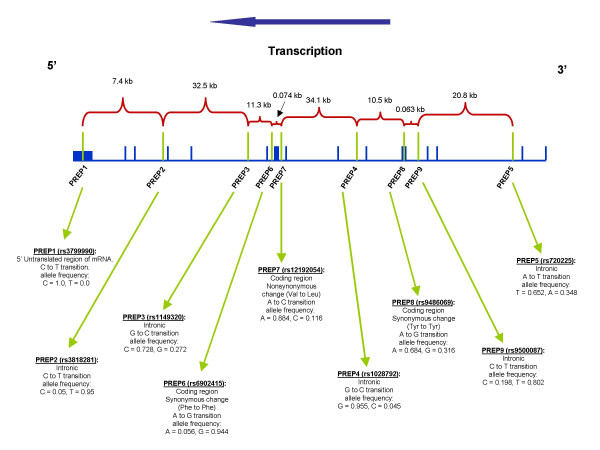
**SNP localization along the PREP gene**. Location of each investigated SNP within the PREP gene with their respective allelic frequencies.

Genomic DNA was extracted using a standardized method (Sambrook et al, 1989) from venous blood samples. Nine single nucleotide polymorphisms (SNP), five intronic, three coding and one located in the 5' UTR, spaced out along the *PREP *gene were investigated using the ABI SNaPshot multiplex reaction and the ABI 3100 automated genetic analyzer (Figure 1). Polymerase chain reaction (PCR) was carried out in a total volume of 15 μl which contained 30 ng genomic DNA, 0.2 μM of each primer, 0.25 mM of each dATP, dNTP, dTTP and dGTP, 0.75 units of Amplitaq Gold DNA polymerase, 1.5 μl of 10× buffer and 25 mM MgCl2. The samples were put through between 30 to 40 cycles of denaturation at 94°C, annealing at specific primer temperatures, elongation at 72°C and a final extension at 72°C. PCR product amplification was verified by running 5 μl of product on a 2% agarose gel. The remaining product was then processed as per the ABI SNaPshot protocol, using primers designed for fluorescent dideoxy nucleotide termination. SNP analysis was carried out on the ABI 3100 genetic analyzer. Genotypes were determined automatically using the Genemapper software (Applied Biosystems).

### Statistical analysis

Hardy-Weinberg (HW) analysis was carried out using the Quick Chi program and those SNPs found to be in disequilibrium were not included in further analyses. Allelic and genotypic frequency distributions were compared between cases and controls, responders versus controls, nonresponders versus controls and finally responders versus nonresponders using a χ^2 ^test and the computer programs SigmaStat and SPSS. Haplotype and linkage disequilibrium (LD) analyses were carried out using both GENECOUNTING and its support programs [17,18]. Our sample had 85% power to detect an association with a variant that is present in 35% of the general population and that doubles the likelihood of lithium response, assuming a type I error no greater than 5%.

## Results

Table 2 outlines the genotypic and allelic distribution and respective χ^2 ^values for the 8 loci investigated; the *PREP3 *SNP was disregarded because it was in HW disequilibrium. Upon completion of analyses several positive results were found prior to correction for multiple testing, interestingly only when the lithium responder subgroup was included in the analysis. When comparing cases and controls the *PREP*6 GG genotype was significantly associated with BD (p = 0.026). This genotype was also found to be significantly associated with response when LiR were compared to LiNR (p = 0.040) or to controls (p = 0.003). Furthermore, in the comparison of responders with controls, the G allele was significantly associated with LiR (p = 0.011).

**Table 2:** Genotypic and Allelic Frequencies for Eight SNPs Analyzed

SNP	Allele	Genotype	Status			
			Cases	Controls	LiR	LiNR

PREP1	C		424 (1,0)	182 (1,0)	155 (1,0)	54 (1,0)
	T		0	0	0	0
		CC	212 (1,0)	91 (1,0)	310 (1,0)	108 (1,0)
		CT	0	0	0	0
		TT	0	0	0	0

PREP2	C		9 (0,021)	21 (0,111)	6 (0,019)	3 (0,027)
	T		429 (0,979)	169 (0,889)	316 (0,981)	109 (0,973)
		CC	1 (0,005)	0	0	1 (0,018)
		CT	7 (0,032)	21 (0,221)	6 (0,037)	1 (0,018)
		TT	211 (0,963)	74 (0,779)	155 (0,963)	54 (0,964)

PREP4	G		391 (0,968)	142 (0,922)	283 (0,969)	102 (0,962)
	C		13 (0,032)	12 (0,078)	9 (0,031)	4 (0,038)
		CC	2 (0,01)	2 (0,026)	1 (0,007)	1 (0,019)
		GC	9 90,045)	8 (0,104)	7 (0,048)	2 (0,038)
		GG	191 (0,946)	67 (0,87)	138 (0,945)	50 (0,943)

PREP5	A		123 (0,324)	73 (0,397)	97 (0,354)	26 (0,26)
	T		257 (0,676)	111 (0,603)	177 (0,646)	74 (0,74)
		AA	20 (0,105)	21 (0,228)	17 (0,124)	3 (0,060)
		AT	82 (0,432)	31 (0,337)	62 (0,453)	20 (0,040)
		TT	88 (0,463)	40 (0,435)	58 (0,423)	27 (0,54)

PREP6	A		28 (0,065)	8 (0,039)	18 (0,060)	10 (0,089)
	G		404 (0,935)	198 (0,961)	284 (0,940)	102 (0,911)
		AA	1 (0,005)	0	1 (0,007)	0
		AG	26 (0,120)	8 (0,078)	16 (0,106)	10 (0,179)
		GG	189 (0,875)	95 (0,922)	134 (0,887)	46 (0,821)

PREP7	A		345 (0,88)	148 (0,892)	221 (0,877)	108 (0,885)
	C		47 (0,12)	18 (0,108)	31 (0,123)	14 (0,115)
		AA	153 (0,781)	66 (0,795)	97 (0,77)	49 (0,803)
		AC	39 (0,199)	16 (0,193)	27 (0,214)	10 (0,164)
		CC	4 (0,020)	1 (0,012)	2 (0,016)	2 (0,033)

PREP8	A		282 (0,681)	123 (0,691)	174 (0,654)	95 (0,720)
	G		132 (0,319)	55 (0,309)	95 (0,346)	37 (0,28)
		AA	98 (0,473)	45 (0,506)	58 (0,436)	34 (0,515)
		AG	86 (0,415)	31 (0,371)	58 (0,436)	27 (0,409)
		GG	23 (0,111)	11 (0,124)	17 (0,128)	5 (0,076)

PREP9	C		81 (0,193)	38 (0,209)	54 (0,20)	26 (0,194)
	T		339 (0,807)	144 (0,791)	216 (0,80)	108 (0,806)
		CC	7 (0,033)	7 (0,077)	5 (0,037)	2 (0,03)
		CT	67 (0,319)	24 (0,264)	44 (0,326)	22 (0,328)
		TT	136 (0,648)	60 (0,659)	86 (0,637)	43 (0,642)

For *PREP*8 both genotypic and allelic associations were found to be significantly associated with lithium response (GA, χ^2 ^= 7.008, df = 2, p = 0.03; A, p = 0.012). *PREP*9 provided significant genotypic and allelic associations in the following comparisons; LiR vs. controls (TT, χ^2 ^= 6.234, df = 2, p = 0.044; T, p = 0.035) and LiR vs. LiNR (TT, χ^2 ^= 7.664, df = 2, p = 0.022; T, p = 0.019). However, after correcting for multiple testing (Bonferroni correction with 36 comparisons), these results became nonsignificant. We did not find any association between age of onset or sex with genotypic or allelic presentation (data not shown).

Estimated haplotype frequencies demonstrated no significant differences in any of the comparisons. Significant linkage disequilibrium was found for most marker pairings (*PREP*4/*PREP*7; D' = 0.539, p = 0.0000; *PREP*8/*PREP*5; D' = 0.695, p = 0.0000; *PREP*8/*PREP*7; D' = 0.372, p = 0.00901; *PREP*4/*PREP*6; D' = 0.234, p = 0.03160).

## Discussion

The phosphoinositide pathway has garnered much interest as a potential system housing genes involved in the etiology of BD. This is mostly in part due to the inhibitory effects of lithium on key enzymes involved in the recycling and the de novo synthesis of free inositol in the cell. A novel association between this pathway and *PREP*, a serine protease involved in neuropeptide breakdown has been reported recently. Williams and collaborators in a series of experiments were able to provide evidence towards the existence of a common mode of action for lithium (Li), valproate (VPA) and carbamazepine (CBZ) implicating also PREP [9]. Their conclusions stem from the effects seen of each drug on the plasticity of growing neurons due to their inhibition of growth cone collapse and an augmentation of the growth cone area [9]. This group had previously characterized a lithium resistant Dictyostelium mutant, with high levels of IP3 and lacking the *PREP *gene [19]. With these mutants it demonstrated that the depletion of inositol is essential for the prophylactic effect of Li. This group was able to extend the inositol depletion requirement to include the function of VPA and CBZ, further suggesting a role of *PREP *in the phosphoinositide pathway and in the efficacy of these drugs [9]. The substantiated support for the possible involvement of *PREP *in the pathophysiology of BD [7-9,19] makes it a likely candidate for investigation. In this study we looked at nine SNPs located along the *PREP *gene for association between lithium response and BD and found no differences when comparing genotypic or allelic distribution between bipolar patients and controls or between responders and nonresponders to lithium treatment following correction for multiple testing.

As it is always the case with negative association studies, it is possible that our negative results are also a consequence of methodological limitations of this study, such as reduced sample power to detect association with variants accounting for small effect sizes or limited information content of the variants investigated. In this regard, further studies using larger samples and investigating additional loci would be of interest. Moreover, expression studies could also be performed looking at the levels of the *PREP *protein in patients with and without good response to lithium treatment.

## Conclusion

In summary our results, albeit negative, provide an initial look into the variation of a gene involved in the phospoinositide pathway and that has been shown to play a role in the mode of action of three common BD treatments; Li, VPA and CBZ. The *PREP *gene is also housed in a region of linkage for BD, which garners additional interest to determine its role in the etiology of BD.

## Competing interests

The author(s) declare that they have no competing interests.

## Authors' contributions

FM performed the molecular genetic studies, carried out analysis and drafted the manuscript. FM and GT designed the experiment. AS, GT and MA helped in drafting the manuscript. MA and PG carried out psychiatric evaluations and were responsible for sample collection. GR performed the DNA extractions. All authors read and approved the manuscript.

## Pre-publication history

The pre-publication history for this paper can be accessed here:


